# A Wide Circuit

**Published:** 1869-03

**Authors:** 


A Wide Circuit.—One of the reporters of the *Glasgow Herald*, in a visit to Malin Head, the northernmost point of Ireland, has fallen in with a medical man whose practice embraces an area of fifty-seven square miles, having a population of 10,000: "Every family in that vast tract," says the writer, "having a claim upon his services, one was quite prepared to hear that often for forty-eight hours together he could not find time either to eat or sleep."

[This is equal to some Dental practitioners we have heard of.—Ed. Reg.]

